# The Persisting Significance of Oligoclonal Bands in the Dawning Era of Kappa Free Light Chains for the Diagnosis of Multiple Sclerosis

**DOI:** 10.3390/ijms19123796

**Published:** 2018-11-29

**Authors:** Philipp Schwenkenbecher, Franz Felix Konen, Ulrich Wurster, Konstantin Fritz Jendretzky, Stefan Gingele, Kurt-Wolfram Sühs, Refik Pul, Torsten Witte, Martin Stangel, Thomas Skripuletz

**Affiliations:** 1 Clinical Neuroimmunology and Neurochemistry, Department of Neurology, Carl-Neuberg-Str. 1, 30625 Hannover, Germany; schwenkenbecher.philipp@mh-hannover.de (P.S.); Konen.Felix@mh-hannover.de (F.F.K.); wurster.ulrich@mh-hannover.de (U.W.); Jendretzky.Konstantin@mh-hannover.de (K.F.J.); Gingele.Stefan@mh-hannover.de (S.G.); Suehs.Kurt-Wolfram@mh-hannover.de (K.-W.S.); Stangel.Martin@mh-hannover.de (M.S.); 2 Department of Neurology, University Clinic Essen, Hufelandstraße 55, 45147 Essen, Germany; Refik.Pul@uk-essen.de; 3 Department of Clinical Immunology and Rheumatology, Carl-Neuberg-Str. 1, 30625 Hannover, Germany; Witte.Torsten@mh-hannover.de

**Keywords:** multiple sclerosis, clinically isolated syndrome, McDonald criteria, kappa free light chains, oligoclonal bands, polyacrylamide gel

## Abstract

The latest revision of the McDonald criteria of 2017 considers the evidence of an intrathecal immunoglobulin (IgG) synthesis as a diagnostic criterion for dissemination in time in multiple sclerosis. While the detection of oligoclonal bands is considered as the gold standard, determination of kappa free light chains might be a promising tool as a less technically demanding and cost saving method. However, data on the direct comparison between kappa free light chains and oligoclonal bands are limited and no study to date has used the highly sensitive method of polyacrylamide gels with consecutive silver staining for the demonstration of oligoclonal bands. Furthermore, the impact of the revised McDonald criteria of 2017 on the role of kappa free light chains as a biomarker has not been investigated. Nephelometry was used to determine kappa free light chains in cerebrospinal fluid (CSF) and serum from 149 patients with their first demyelinating event between 2010 and 2015. Clinical data, kappa free light chains, and oligoclonal band status were compared at the time of initial diagnosis and after follow-up to identify converters from clinically isolated syndrome to multiple sclerosis. An elevated kappa free light chain index (>5.9) was found in 79/83 patients (95%) with multiple sclerosis diagnosed at baseline, slightly less frequent than oligoclonal bands (98.8%). 18/25 (72%) patients who converted from clinically isolated syndrome to multiple sclerosis showed an elevated kappa free light chain index compared to 20/25 (80%) patients with positive oligoclonal bands. In patients with stable clinically isolated syndrome 7/41 (17%) displayed an elevated kappa free light chain index against 11/41 (27%) oligoclonal band positive patients. Only two patients with stable clinically isolated syndrome showed an elevated kappa free light chain index but were oligoclonal bands negative. In conclusion, determination of the kappa free light chain index is a promising diagnostic approach to assess intrathecal immunoglobulin synthesis in multiple sclerosis. Nevertheless, oligoclonal bands are highly prevalent in multiple sclerosis and can detect an intrathecal synthesis of IgG even when the kappa free light chain index is below the threshold. We consider sequential use of both methods as reasonable.

## 1. Introduction

The detection of an intrathecal immunoglobulin (IgG) synthesis in the form of oligoclonal IgG bands is considered to be the most important laboratory evidence of multiple sclerosis as a chronic inflammatory disease [1]. Before the revision of McDonald criteria for multiple sclerosis diagnosis of 2010, lack of evidence for dissemination in space could be substituted by the detection of oligoclonal bands [2]. As the minimum number of magnetic resonance imaging (MRI) lesions required to demonstrate dissemination in space was reduced in the revision of McDonald criteria of 2010, oligoclonal bands were omitted from the diagnostic criteria except for primary progressive multiple sclerosis [1]. Since numerous studies identified oligoclonal bands as an independent risk factor for conversion from clinically isolated syndrome to multiple sclerosis, the latest revision of McDonald criteria of 2017 rejuvenated oligoclonal bands as a criterion to demonstrate dissemination in time [3]. Although considered as the gold standard for detection of an intrathecal IgG synthesis, determination of oligoclonal bands requires methodological expertise and may be rater-dependent [4,5]. Measurement of kappa free light chains (KFLC) was suggested as an alternative procedure [5,6]. In analogy to immunoglobulins, the intrathecally produced fraction of KFLC and thus the evidence of B-cell activity in cerebrospinal fluid (CSF) can be determined by the KFLC index [6], the most commonly suggested method for quantitation of KFLC synthesis. Uncertainties in result calculation, the definition of comparable cut-offs, and the dependence on the specific method used have so far hindered the determination of KFLC measurement in routine diagnostic work-up [5]. Especially, KFLC measurements have often been compared to less sensitive methods of oligoclonal band determination. In the present study, the value of kappa free light chains was evaluated against separation of oligoclonal bands on polyacrylamide gels with consecutive silver staining in patients diagnosed using the new McDonald criteria of 2017.

## 2. Results

This study compromises a total of 149 patients with paired CSF and serum samples. 83 of these patients fulfilled the multiple sclerosis diagnostic criteria according to McDonald 2017 at presentation. 25 patients with clinically isolated syndrome converted to multiple sclerosis because of a new relapse or new MRI lesions, while 41 patients remained as stable clinically isolated syndrome during a follow up of a median of 50 months (range 24–87 months). Clinical characteristics are presented in Table 1.

Demographic and clinical characteristics of patients diagnosed with multiple sclerosis according to the McDonald criteria of 2017, clinically isolated syndrome who converted to multiple sclerosis during follow-up, and stable clinically isolated syndrome.

### 2.1. Oligoclonal Bands in Relation to KFLC Indices in All Patients

Positive CSF oligoclonal bands were found in 113 of all 149 patients (76%). 102 of 113 patients (90%) with oligoclonal bands showed also an elevated KFLC index >5.9 (median 63.6; range 5.95–539.7). In contrast, only 2/36 patients (6%) without oligoclonal bands expressed an elevated KFLC index. These patients expressed low values of 6.75 and 7.83.

**Figure 1:**
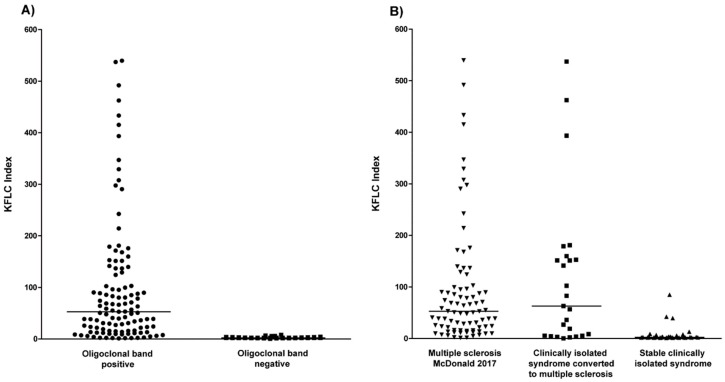
Kappa free light chain (KFLC) index in different cohorts. (**A**) The KFLC index defined as (KFLC in cerebrospinal fluid (CSF)/KFLC in serum) divided by albumin quotient (albumin in CSF/albumin in serum) in all patients with positive (dots) and negative oligoclonal bands (squares). (**B**) The KFLC index in patients diagnosed with multiple sclerosis according to the McDonald criteria of 2017 (down arrowhead), clinically isolated syndrome who converted to multiple sclerosis during follow-up (squares), and stable clinically isolated syndrome (up arrowhead).

Patients with oligoclonal bands showed significantly higher KFLC index levels compared to patients with negative oligoclonal bands (*p* < 0.0001; Figure 1A).

### 2.2. Oligoclonal Bands in Multiple Sclerosis and Clinically Isolated Syndrome

Oligoclonal bands were positive in 82/83 (98.8%) of patients with multiple sclerosis. In patients with clinically isolated syndrome who converted to multiple sclerosis, oligoclonal bands were found in 20/25 (80%) cases. In contrast, only 11/41 (27%) of patients with stable clinically isolated syndrome showed positive oligoclonal bands.

### 2.3. KFLC Indices in Multiple Sclerosis and Clinically Isolated Syndrome

An elevated KFLC index as defined by a value of >5.9 was found in 79/83 (95%) of patients with multiple sclerosis (median 55.2; range 5.95–539.7). In patients with clinically isolated syndrome who converted to multiple sclerosis, an elevated KFLC index was found in 18/25 (72%) cases (median 146.4; range 8.6–539.1). In contrast, only 7/41 (17%) of patients with stable clinically isolated syndrome showed an elevated KFLC index (median 13.1; range 6.75–84.9).

The KFLC index values did not differ between patients with multiple sclerosis and patients with clinically isolated syndrome who converted to multiple sclerosis (*p* = 0.98). As shown in Figure 1B, the KFLC index values were significantly lower in patients with stable clinically isolated syndrome as compared with multiple sclerosis patients (*p* < 0.0001) and clinically isolated syndrome patients who converted to multiple sclerosis (*p* < 0.0001).

### 2.4. KFLC Indices Compared to Oligoclonal Bands in Patients with Multiple Sclerosis

An elevated KFLC index was found in 79 of 82 (96.3%) patients with multiple sclerosis and positive oligoclonal bands. Two of the three patients with a normal KFLC index expressed a low number of oligoclonal bands (2–3 bands) and KFLC index values of 1.8 and 1.61. The only patient with multiple sclerosis and negative oligoclonal bands did not display an elevated KFLC index as well.

### 2.5. KFLC Indices Compared to Oligoclonal Bands in Patients with Clinically Isolated Syndrome Who Converted to Multiple Sclerosis

**Figure 2:**
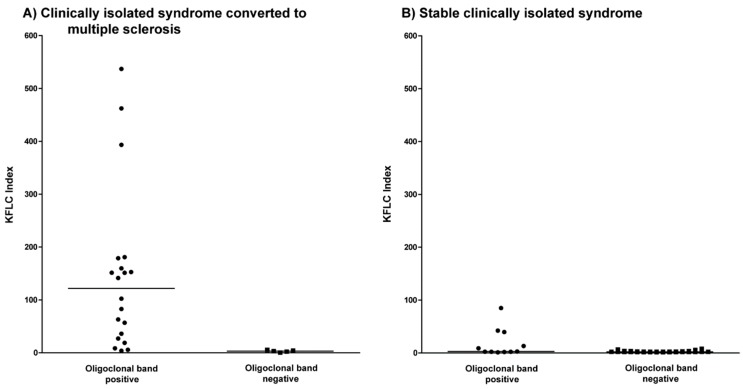
Kappa free light chain (KFLC) index in clinically isolated syndrome. (**A**) The KFLC index defined as (KFLC in CSF/KFLC in serum) divided by albumin quotient (albumin in CSF/albumin in serum) in patients with clinically isolated syndrome who converted to multiple sclerosis during follow-up compared between patients with positive (dots) and negative oligoclonal bands (squares). (**B**) The KFLC index in patients with stable clinically isolated syndrome compared between patients with positive (dots) and negative oligoclonal bands (squares).

A total of 18 of 20 patients (90%) with clinically isolated syndrome who converted to multiple sclerosis and were positive for oligoclonal bands displayed an elevated KFLC index (Figure 2A). One of the two patients with a normal KFLC index expressed a low number of oligoclonal bands (2–3 bands) and a KFLC index value of 5.67. The five patients with clinically isolated syndrome who converted to multiple sclerosis and negative oligoclonal bands likewise did not display an elevated KFLC index.

### 2.6. KFLC Indices Compared to Oligoclonal Bands in Patients with Stable Clinically Isolated Syndrome

An elevated KFLC index was found in 5 of 11 (45%) patients with stable clinically isolated syndrome and positive oligoclonal bands (Figure 2B). Five of the six patients with a normal KFLC index expressed a low number of oligoclonal bands (2–3 bands) and KFLC index values of 2.05, 2.73, 1.85, 1.03, and 2.12.

A total of 2 of 30 patients with stable clinically isolated syndrome and negative oligoclonal bands showed a slightly elevated KFLC index of 7.83 and 6.75.

### 2.7. KFLC Indices Compared to Quantitative IgG Synthesis in Reiber-Graphs

**Figure 3:**
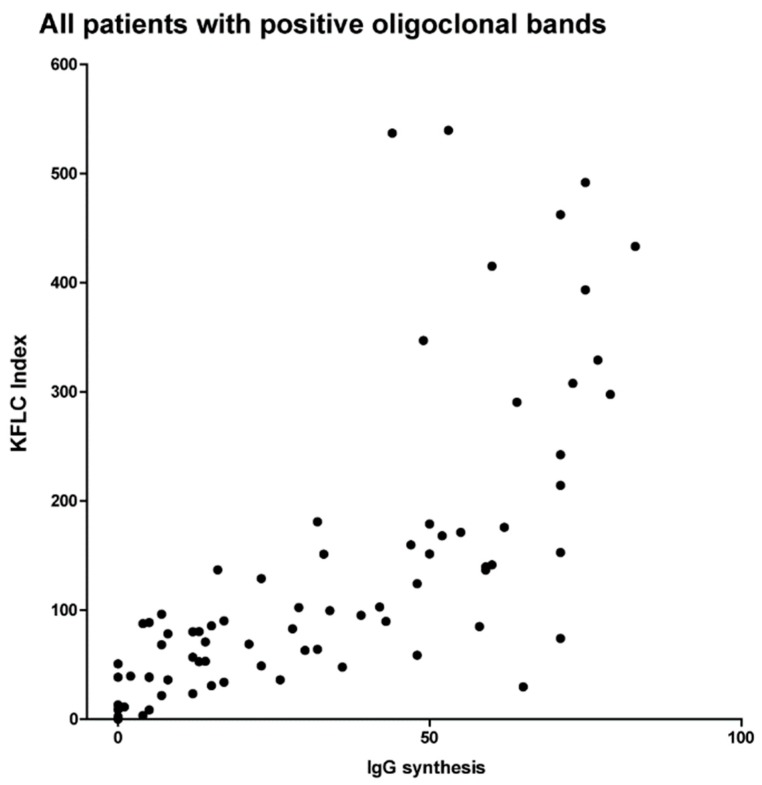
Kappa free light chain indices and quantitative immunoglobulin (IgG) synthesis.

In Figure 3, the expressions of intrathecal IgG synthesis (0–100%) as detected in Reiber-graphs are shown in comparison to the values of the KFLC indices in all patients with positive oligoclonal bands. Although the comparison of percentage values of IgG synthesis in Reiber-graphs and absolute values of KFLC indices do not allow statistical analysis in sense of regression curves and *p*-values, the correlation between increasing intrathecal IgG synthesis and elevated KFLC indices seems evident.

The KFLC index defined as (KFLC in CSF/KFLC in serum) divided by albumin quotient (albumin in CSF/albumin in serum) compared with quantitative immunoglobulin synthesis as defined by Reiber-graphs from 0% to 100%.

## 3. Discussion

The latest revision of the McDonald criteria for multiple sclerosis in 2017 marks a turning point for the significance of CSF diagnostic [3]. While in the previous revision in 2010 the evidence of an intrathecal synthesis of immunoglobulin played no role for the diagnosis of relapsing-remitting multiple sclerosis, oligoclonal bands can now serve as a biomarker to demonstrate dissemination in time [3]. Oligoclonal band determination is considered to be the gold standard to prove intrathecal synthesis of IgG [1,7]. However, several studies have highlighted the convincing performance and low cost of KFLC making them an alternative to oligoclonal bands. In contrast to oligoclonal bands, KFLC may serve as quantitative marker for the intrathecal autoimmune response [7,8,9,10,11,12,13]. There is an ongoing discussion as to whether KFLC measuring should be combined with oligoclonal bands determination or should even replace oligoclonal bands as a first-line test [5,14]. While previous reports employed isoelectric focusing (IEF) with several methods based on agarose IEF, we present the first investigation of CSF-specific oligoclonal bands determined by IEF in polyacrylamide gels with consecutive silver staining [5,14]. Silver staining offers a higher resolution as it detects closely adjacent bands as two separate bands, whereas they merge into a single broader band in other methods in which diffusion after separation is unavoidable [15].

In this study, we analyzed the diagnostic performance of oligoclonal bands determined with the silver staining technique versus KFLC determination in a well-defined real-life cohort of patients who presented with a first demyelinating event. In the cohort of patients who were diagnosed with multiple sclerosis at baseline according to the latest McDonald criteria of 2017, oligoclonal bands were found in all but one patient (98.8%). An elevated KFLC index was found in all but three patients (96.4%) diagnosed with multiple sclerosis and positive oligoclonal bands indicating a promising consistency. However, three patients with positive oligoclonal bands showed a KFLC index below the threshold resulting in a negative result. A similar observation could be made in the cohort of patients with clinically isolated syndrome. Eight patients with clinically isolated syndrome (two patients who converted to multiple sclerosis and six patients with a stable course during follow-up) expressed positive oligoclonal bands but could not be confirmed by an elevated KFLC index (26%). In these patients who failed to express an elevated KFLC index, a low number of oligoclonal bands (in 8/11 patients only 2–3 bands) was found meaning a weak but positive result. The low synthesis of intrathecal IgG production as shown by a low number of bands might explain the negative result of the KFLC index. These findings are in accordance with the observation that higher amounts of an intrathecal IgG synthesis (as shown by using Reiber-graphs) resulted in high values of measured KFLC indices. On the other side, two patients with clinically isolated syndrome whose KFLC index was slightly elevated did not show oligoclonal bands. Both patients suffered from a clinically isolated syndrome with a stable course during follow up.

We confirm once and again that oligoclonal bands are highly sensitive in multiple sclerosis [16] and can detect an intrathecal synthesis of IgG even when the KFLC index is below the threshold. Furthermore, in our cohort we could not identify patients with multiple sclerosis and clinically isolated syndrome who converted to multiple sclerosis during follow up who showed an elevated KFLC index but no oligoclonal bands. These observations underline the higher sensitivity of oligoclonal bands in multiple sclerosis diagnosed according the McDonald criteria of 2017 due to the use of a highly sensitive method. However, if oligoclonal bands or KFLC are used as biomarkers to predict the conversion rate to multiple sclerosis, three times more oligoclonal band positive patients who were KFLC negative than KFLC positive patients who were oligoclonal band negative were found who remained stable during follow-up.

In our study, oligoclonal bands were more sensitive (94%, 102/108) than KFLC index (89%, 97/108) for patients who were diagnosed with multiple sclerosis at baseline or converted to multiple sclerosis during follow-up. On the other hand, oligoclonal bands were less specific (73%, 30/41) than KFLC index (83%, 34/41) in identifying multiple sclerosis in our cohort. This result is in line with previous observations in which KFLC determination has a higher specificity but lower sensitivity compared to oligoclonal bands [4]. Oligoclonal bands have been reported to be found in up to 5% of healthy subjects [17]. Furthermore, infections, paraneoplastic disorders, or other autoimmune diseases of the CNS may provoke a humoral response resulting in positive oligoclonal bands [16]. To date, large studies comparing the diagnostic performance of KFLC with oligoclonal bands in a non-multiple sclerosis cohort are lacking.

Only longer observation times can show whether conversion will occur at a later date. Consequently, if confirmed in a larger population and during longer follow-up periods, the apparent higher specificity in identifying multiple sclerosis patients would be of high importance and a strong argument for determining KFLC index in every clinically isolated syndrome patient. Furthermore, since previous studies demonstrated that the KFLC index also predicts disease progression, the quantification of KFLC might be valuable in guiding therapeutic decisions [18].

Based on rater variability and cost awareness, Crespi and colleagues suggested a sequential approach with KFLC determination as a screening test followed by oligoclonal band detection in positive cases [14]. Due to the presented results, we believe that oligoclonal bands may still be used as a screening method. In our study, we could identify six patients who were either diagnosed with multiple sclerosis at baseline or converted to multiple sclerosis in the course of the disease who revealed negative KFLC index results but showed positive oligoclonal bands. In contrast, we could not identify patients diagnosed with multiple sclerosis who were oligoclonal bands negative but KFLC positive.

The determination of KFLC is definitely a quick and machine-operated technique in comparison to the manual isoelectric focusing and the subsequent, although also highly automated silverstain, with visual evaluation by specially trained personnel. On the other hand, a definite criterion exists for a positive oligoclonal band result with two bands in the CSF only, whereas borderline KFLC index values appear more uncertain and depend heavily on the proper technique used and on the employed evaluation schemes, which are far away from common agreement.

In conclusion, determination of KFLC in the future will be more broadly employed for the determination of immunoglobulin synthesis in multiple sclerosis, but demonstration of properly conducted oligoclonal band determination will continue to be of high value and is the sole parameter recognized by the McDonald criteria of 2017 as a substitute for the dissemination in time.

## 4. Methods

### 4.1. Patients

In this study, we retrospectively investigated paired CSF and serum samples from routine lumbar puncture from patients who presented with symptoms suggestive for a demyelinating disease from 2010 to 2015 at the Department of Neurology of the Hannover Medical School (Hannover, Germany). Patients were divided into three groups: newly diagnosed multiple sclerosis according to the McDonald criteria of 2017, clinically isolated syndrome at presentation who converted to multiple sclerosis during follow-up, and clinically isolated syndrome with a stable course during follow-up. These groups consisted of patients that were previously described [19,20]. Patients were only included when paired CSF and serum samples were available and diagnosis was confirmed at follow-up. This investigation was approved by the institutional ethics committee of the Hannover Medical School (No. 7837_BO_K_2018, 6 April 2018).

### 4.2. CSF and Serum Analytical Procedures

Routine diagnostic work-up was performed in all paired CSF and serum samples in the Neurochemistry Laboratory of the Department of Neurology. After completion of these diagnostic procedures, samples were immediately stored at −80 °C. CSF cell counts were determined microscopically with Fuchs Rosenthal counting chamber. By a Bradford dye-binding procedure, total protein (cut-off = 500 mg/L) was measured in centrifuged CSF samples [21]. Kinetic nephelometry (Beckman Coulter IMMAGE, Brea, CA, USA) was used to determine levels of albumin and IgG, IgA, and IgM. Quantitative intrathecal synthesis of IgG was calculated by the method of Reiber-Felgenhauer [21]. CSF-specific oligoclonal bands were determined by isoelectric focusing on polyacrylamide gels with consecutive silver staining [22]. The concentrations of KFLC in sera and CSF were measured by nephelometry with N Latex FLC kappa Kit (Siemens Healthcare Diagnostics Products GmbH, Erlangen, Germany), according to the manufacturer’s instruction on a BN Prospec analyzer (Siemens Healthcare Diagnostics Products GmbH). Since measuring KFLC concentrations only in CSF or calculating its CSF/serum ratio without reference to blood-CSF barrier function has only limited diagnostic value, the intrathecal fraction of KFLC was determined [6]. To determine the intrathecal fraction of KFLC, the KFLC index was calculated by the formula (KFLC CSF/KFLC serum)/(Albumin CSF/Albumin serum). Values above the empirically defined threshold KFLC index >5.9 were considered as an elevated KFLC index, indicating an intrathecal synthesis of KFLC [12]. The Neurochemistry Laboratory of the Department of Neurology (Hannover, Germany) participates regularly in the external INSTAND survey program for analytic methods quality control [23].

### 4.3. Statistical Analysis

For statistical analysis, GraphPad Prism (La Jolla, CA, USA; version 5.02) was used. Values are presented as median and range. Fisher’s exact test was performed for categorical data, and Mann-Whitney U-Test for independent values. The D’Agostino-Pearson normality test was used to asses for normal distribution of values. *p*-values < 0.05 were considered as statistically significant.

## Figures and Tables

**Table 1:** Patient demographic and clinical characteristics.

Characteristics	Multiple Sclerosis McDonald 2017	Clinically Isolated Syndrome Converted to Multiple Sclerosis	Stable Clinically Isolated Syndrome
Females, *n* (%)	57/83 (69%)	16/25 (64%)	27/41 (66%)
Age, years (range)	31 (17–73)	33 (17–73)	37 (16–54)
Optic neuritis, *n* (%)	34/83 (41%)	10/25 (40%)	38/41 (93%)
Paresis/sensory symptoms, *n* (%)	13/83 (16%)	3/25 (12%)	2/41 (5%)
Brainstem symptoms, *n* (%)	14/83 (17%)	4/25 (16%)	1/41 (2%)
Spinal cord symptoms, *n* (%)	15/83 (18%)	8/25 (32%)	0
Polysymptomatic, *n* (%)	7/83 (8%)	0	0

## References

[1] Stangel M., Fredrikson S., Meinl E., Petzold A., Stuve O., Tumani H. (2013). The utility of cerebrospinal fluid analysis in patients with multiple sclerosis. Nat. Rev. Neurol..

[2] Petzold A. (2013). Intrathecal oligoclonal IgG synthesis in multiple sclerosis. J. Neuroimmunol..

[3] Thompson A.J., Banwell B.L., Barkhof F., Carroll W.M., Coetzee T., Comi G., Correale J., Fazekas F., Filippi M., Freedman M.S. (2017). Diagnosis of multiple sclerosis: 2017 revisions of the McDonald criteria. Lancet Neurol..

[4] Senel M., Tumani H., Lauda F., Presslauer S., Mojib-Yezdani R., Otto M., Brettschneider J. (2014). Cerebrospinal fluid immunoglobulin kappa light chain in clinically isolated syndrome and multiple sclerosis. PLoS ONE.

[5] Susse M., Hannich M., Petersmann A., Zylla S., Pietzner M., Nauck M., Dressel A. (2018). Kappa free light chains in cerebrospinal fluid to identify patients with oligoclonal bands. Eur. J. Neurol..

[6] Presslauer S., Milosavljevic D., Huebl W., Aboulenein-Djamshidian F., Krugluger W., Deisenhammer F., Senel M., Tumani H., Hegen H. (2016). Validation of kappa free light chains as a diagnostic biomarker in multiple sclerosis and clinically isolated syndrome: A multicenter study. Mult. Scler..

[7] Bourahoui A., De Seze J., Guttierez R., Onraed B., Hennache B., Ferriby D., Stojkovic T., Vermersch P. (2004). CSF isoelectrofocusing in a large cohort of MS and other neurological diseases. Eur. J. Neurol..

[8] Fischer C., Arneth B., Koehler J., Lotz J., Lackner K.J. (2004). Kappa free light chains in cerebrospinal fluid as markers of intrathecal immunoglobulin synthesis. Clin. Chem..

[9] Kaplan B., Aizenbud B.M., Golderman S., Yaskariev R., Sela B.A. (2010). Free light chain monomers in the diagnosis of multiple sclerosis. J. Neuroimmunol..

[10] Krakauer M., Schaldemose Nielsen H., Jensen J., Sellebjerg F. (1998). Intrathecal synthesis of free immunoglobulin light chains in multiple sclerosis. Acta Neurol. Scand..

[11] Meinl E., Derfuss T., Krumbholz M., Probstel A.K., Hohlfeld R. (2011). Humoral autoimmunity in multiple sclerosis. J. Neurol. Sci..

[12] Presslauer S., Milosavljevic D., Brucke T., Bayer P., Hubl W. (2008). Elevated levels of kappa free light chains in CSF support the diagnosis of multiple sclerosis. J. Neurol..

[13] Rudick R.A., French C.A., Breton D., Williams G.W. (1989). Relative diagnostic value of cerebrospinal fluid kappa chains in MS: Comparison with other immunoglobulin tests. Neurology.

[14] Crespi I., Sulas M.G., Mora R., Naldi P., Vecchio D., Comi C., Cantello R., Bellomo G. (2017). Combined use of Kappa Free Light Chain Index and Isoelectrofocusing of Cerebro-Spinal Fluid in Diagnosing Multiple Sclerosis: Performances and Costs. Clin. Lab..

[15] Wurster U., Zettl U.K., Lehmitz R., Mix E. (2005). Elektrophoreseverfahren—Nachweis und Bedeutung von oligoklonalen Banden. Klinische Liquordiagnostik.

[16] Link H., Huang Y.M. (2006). Oligoclonal bands in multiple sclerosis cerebrospinal fluid: An update on methodology and clinical usefulness. J. Neuroimmunol..

[17] Wurster U., Stachan R., Windhagen A., Petereit H.F., Leweke F.M. (2006). Reference values for standard cerebrospinal fluid examinations in multiple sclerosis. Results from 99 healthy volunteers. Mult Scler.

[18] Rathbone E., Durant L., Kinsella J., Parker A.R., Hassan-Smith G., Douglas M.R., Curnow S.J. (2018). Cerebrospinal fluid immunoglobulin light chain ratios predict disease progression in multiple sclerosis. J. Neurol. Neurosurg. Psychiatry.

[19] Schwenkenbecher P., Sarikidi A., Bonig L., Wurster U., Bronzlik P., Suhs K.W., Pul R., Stangel M., Skripuletz T. (2017). Clinically Isolated Syndrome According to McDonald 2010: Intrathecal IgG Synthesis Still Predictive for Conversion to Multiple Sclerosis. Int. J. Mol. Sci..

[20] Schwenkenbecher P., Sarikidi A., Wurster U., Bronzlik P., Suhs K.W., Raab P., Stangel M., Pul R., Skripuletz T. (2016). McDonald Criteria 2010 and 2005 Compared: Persistence of High Oligoclonal Band Prevalence Despite Almost Doubled Diagnostic Sensitivity. Int. J. Mol. Sci..

[21] Reiber H. (1998). Cerebrospinal fluid—Physiology, analysis and interpretation of protein patterns for diagnosis of neurological diseases. Mult. Scler..

[22] Andersson M., Alvarez-Cermeno J., Bernardi G., Cogato I., Fredman P., Frederiksen J., Fredrikson S., Gallo P., Grimaldi L.M., Gronning M. (1994). Cerebrospinal fluid in the diagnosis of multiple sclerosis: A consensus report. J. Neurol. Neurosurg. Psychiatry.

[23] Reiber H. (1995). External quality assessment in clinical neurochemistry: Survey of analysis for cerebrospinal fluid (CSF) proteins based on CSF/serum quotients. Clin. Chem..

